# Supplementary material for: 3D-Printed Polylactic Acid/Lignin Films with Great Mechanical Properties and Tunable Functionalities towards Superior UV-Shielding, Haze, and Antioxidant Properties
Source: Polymers (Basel). 2023 Jun 24;15(13):2806. doi: 10.3390/polym15132806 (PMC10346654; doi:10.3390/polym15132806)
Supplement: Supplementary file 1 [file polymers-15-02806-s001.zip › polymers-2441168-supplementary.docx]

Article

3D−Printed Polylactic Acid/Lignin Films with Great Mechanical Properties and Tunable Functionalities towards Superior UV−Shielding, Haze, and Antioxidant Properties

| **Citation:** Ye, H.; He, Y.; Li, H.;  You, T.; Xu, F. 3D−Printed Polylactic Acid/Lignin Films with Great  Mechanical Properties and Tunable  Functionalities towards Superior UV−Shielding, Haze, and  Antioxidant Properties. *Polymers* **2023**, *15*, 2806. https://doi.org/ 10.3390/polym15132806  Academic Editor: Rong-Ho Lee  Received: 24 May 2023  Revised: 15 June 2023  Accepted: 21 June 2023  Published: 24 June 2023  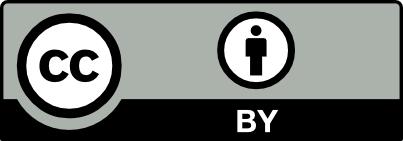  **Copyright:** © 2023 by the authors. Licensee MDPI, Basel, Switzerland. This article is an open access article distributed under the terms and conditions of the Creative Commons Attribution (CC BY) license (https://creativecommons.org/licenses/by/4.0/). |
| --- |

Haichuan Ye ^1,2^, Yuan He ^1,2^, Haichao Li ^1,2^, Tingting You ^1,2,^* and Feng Xu ^1,2,3,^*

^1^ Beijing Key Laboratory of Lignocellulosic Chemistry, Beijing Forestry University, Beijing 100083, China; haichuan.ye@foxmail.com (H.Y.); hy941813@163.com (Y.H.); lihaichao96@bjfu.edu.cn (H.L.)

^2^ Engineering Research Center of Forestry Biomass Materials and Energy, Ministry of Education, Beijing Forestry University, Beijing 100083, China

^3^ Shandong Key Laboratory of Paper Science & Technology, Qilu University of Technology,
Jinan 250353, China

***** Correspondence: youtingting0928@bjfu.edu.cn (T.Y.); xfx315@bjfu.edu.cn (F.X.)

**Supplementary Materials**


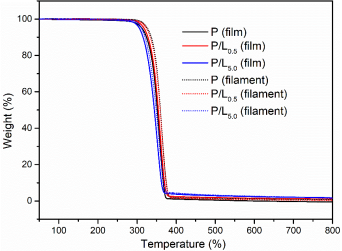


**Figure S1**. TG curves of 3D printed PLA/lignin films and PLA/lignin filaments
